# Combining data acquisition modes in liquid-chromatography–tandem mass spectrometry for comprehensive determination of acylcarnitines in human serum

**DOI:** 10.1007/s11306-022-01916-5

**Published:** 2022-07-21

**Authors:** D. Luque-Córdoba, M. Calderón-Santiago, F. Priego-Capote

**Affiliations:** 1 Department of Analytical Chemistry, University of Córdoba, Annex Marie Curie Building, Campus of Rabanales, Córdoba, Spain; 2 Nanochemistry University Institute (IUNAN), University of Córdoba, Campus of Rabanales, Córdoba, Spain; 3 Maimónides Institute of Biomedical Research (IMIBIC), Reina Sofía University Hospital, University of Córdoba, Córdoba, Spain; 4 Consortium for Biomedical Research in Frailty & Healthy Ageing, Carlos III Institute of Health, CIBERFES, Madrid, Spain

**Keywords:** Acylcarnitines, Serum, SPE–LC–MS/MS, Data-independent acquisition, Data-dependent acquisition, Multiple reaction monitoring

## Abstract

Acylcarnitines (ACs) are metabolites involved in fatty acid β-oxidation and organic acid metabolism. Metabolic disorders associated to these two processes can be evaluated by determining the complete profile of ACs. In this research, we present an overall strategy for identification, confirmation, and quantitative determination of acylcarnitines in human serum. By this strategy we identified the presence of 47 ACs from C2 to C24 with detection of the unsaturation degree by application of a data-independent acquisition (DIA) liquid chromatography–tandem mass spectrometry (LC–MS/MS) method. Complementary, quantitative determination of ACs is based on a high-throughput and fully automated method consisting of solid-phase extraction on-line coupled to LC–MS/MS in data-dependent acquisition (DDA) to improve analytical features avoiding the errors associated to sample processing. Quantitation limits were at pg mL^–1^ level, the intra-day and between-day variability were below 15–20%, respectively; and the accuracy, expressed as bias, was always within ± 25%. The proposed method was tested with 40 human volunteers to determine the relative concentration of ACs in serum and identify predominant forms. Significant differences were detected by comparing the ACs profile of obese versus non-obese individuals.

**Supplementary Information:**

The online version contains supplementary material available at 10.1007/s11306-022-01916-5.

## Introduction

L-carnitine is a quaternary amine acquired by humans mainly through diet, although it is also biosynthesized from lysine and methionine in the liver and kidneys. Carnitine is an essential compound in the metabolism of mammals because of its role in the transport of fatty acids across the mitochondrial membrane by esterification with acyl-coenzyme A derivatives to produce acylcarnitines (ACs) for subsequent β-oxidation of fatty acids (Fritz & Yue, 1963).

An impaired β-oxidation and mitochondrial dysfunction can lead to elevated concentration of ACs in serum (Shah et al., 2010), which has been associated to obesity, insuline resistance, type 2 diabetes and metabolic syndrome (Mihalik et al., 2010; Roberts et al., 2014), and an increased risk of cardiovascular disease (Guasch-Ferré et al., 2016). Other studies have associated these disorders to relative changes in the ACs profile. Thus, Guasch-Ferré et al. (2019) determined that short- and long-chain ACs (SCACs and LCACs, respectively) are significantly associated with type 2 diabetes risk in cardiovascular disease patients. In the same line, Mihalik et al. (2010) pointed out an accumulation of LCACs in obese and type 2 diabetes patients that arise from the activity in the initial rounds of fatty acid β-oxidation. In another study, Bene et al. (2013) reported changes in the levels of SCACs, LCACs and medium-chain ACs (MCACs) in patients with type 1 and 2 diabetes, and metabolic syndrome. In contrast, other pathologies such as the chronic fatigue syndrome has been associated to deficiency in AC levels (Kuratsune et al., 1994). With these premises, the involvement of ACs in the metabolism of fatty acids suggests they could be considered markers to monitor metabolic disorders, although the diet can influence the relative levels of some species (Wedekind et al., 2020).

A multitude of analytical methods have been proposed to determine ACs in human blood. All protocols implement a protein precipitation step using mainly acetonitrile (ACN) (Ferrer et al., 2007; Peng et al., 2013), methanol (MeOH) (Giesbertz et al., 2015; Gucciardi et al., 2012; Rizzo et al., 2014) or isopropanol (IPA) (M. Wang et al., 2019a) prior to typical steps such as solid phase extraction (SPE) and derivatization to improve detection. SPE is mainly carried out with ion exchange sorbents based on weak cation exchange (WCX) (Horvath et al., 2010) and mixed strong cation exchange (MCX) mechanisms (Maeda et al., 2007, 2008; Minkler et al., 2015). In some cases, SPE steps have been on-line coupled to liquid chromatography separation by using column switching units with clean-up purposes (Ghoshal et al., 2005; Minkler et al., 2015; Morand et al., 2013). However, previous deproteinization do not allow full automation of a given method.

Mass spectrometry is the most widely used detection technique for determination of ACs, particularly tandem mass spectrometry (MS/MS) with electrospray ionization. Derivatization is a common step in protocols for determination of ACs, frequently based on esterification with *n*-butanol (Ferrer et al., 2007; Gucciardi et al., 2012; Rizzo et al., 2014), which allows improving ionization efficiency and overcoming isobaric interferents such as dicarboxylic AC derivatives (Giesbertz et al., 2015). However, some authors pointed out that butylation would not be the most appropriate derivatization process, since the ester bond could be hydrolyzed, thereby increasing the content of free carnitine (Kivilompolo et al., 2013; Minkler et al., 2015). Moreover, the addition of the butyl group implies an increase in the retention time in chromatographic methods that use reversed phase columns. On the other hand, Minkler et al. (2015) proposed the use of pentafluorophenacyl trifluoromethanesulfonate (PFP TFMS) to avoid problems derived from partial hydrolysis.

Detection of underivatized ACs is one other alternative in positive ionization mode (ESI +) (Giesbertz et al., 2015; Gucciardi et al., 2012). The quaternary amine has a permanent positive charge, while the negative charge strictly depends on the pH since protonation of the carboxylic group occurs at a low pH (within 3–5). There are methods based on direct infusion in ESI–MS/MS to screen the ACs profile for detection of pathologies in newborns (Turgeon et al., 2008). However, these methods do not allow isomer discrimination, thus leading to false positives detection (Abdenur et al., 1998). To avoid these interferences (isobaric and isomeric), LC–MS/MS must be used (Minkler et al., 2015). Structurally, the analogous character of these compounds supports a common fragmentation pattern for linear ACs, which can be used to design detection methods based on different acquisition modes.

According to the described state-of-the-art, an overall strategy for comprehensive qualitative and quantitative determination of ACs in human serum is proposed by the present research. The approach combines data independent and dependent acquisition modes (DIA and DDA, respectively) for identification, confirmation, and quantitation of ACs. The aims of the proposed method are: (i) to offer an analytical tool to characterize the ACs profile by discriminating species according to the length of hydrocarbon chains (SCACs, MCACs and LCACs) and unsaturation level (saturated, mono- and polyunsaturated species); (ii) to provide a complete automated quantitative method involving direct analysis to minimize human intervention.

## Material and methods

### Reagents

Chromatographic grade ACN and MeOH were purchased from Scharlab (Barcelona, Spain). Mass spectrometry grade ammonium formate, formic acid (FA) and ammonia solution (35% v/v) were from Fisher scientific (Madrid, Spain). Milli-Q-water was generated by a Simplicity® UV Millipore equipment (Burlington, MA, USA). Isotopically labelled ACs used as internal standards (IL-ISs) –namely, acetyl-L-carnitine-d_3_ (C2-d_3_), butyryl-L-carnitine-d_3_ (C4-d_3_) hexanoyl-L-carnitine-d_3_ (C6-d_3_), decanoyl-L-carnitine-d_3_ (C10-d_3_), lauroyl-L-carnitine-d_9_ (C12-d_9_), miristoyl-L-carnitine-d_9_ (C14-d_9_) and palmitoyl-L-carnitine-d_3_ (C16-d_3_) were acquired from Sigma-Aldrich (St. Louis, MO, USA). Stock solutions of IL-IS were prepared separately in MeOH at 100 µg mL^–1^. A multistandard IL-IS solution was prepared at 2 µg mL^–1^ in ACN, and a working solution was obtained by diluting the multistandard solution at 400 ng mL^–1^.

### Sample collection

Blood samples from forty volunteers were extracted in fasting state into adequate plastic plasma Vacutainer® tubes (Becton Dickinson, East Rutherford, NJ, USA) in compliance with World Medical Association Declaration of Helsinki (2004) guidelines. The Ethics Committee of the Reina Sofia University Hospital (Cordoba, Spain) approved the study, and a written informed consent was signed by every individual before inclusion into the study. General characteristics of the included patients are summarized in Table 1. Comorbidities were discarded in normal weight adults, while obese individuals were not diagnosed with other pathologies not associated to obesity. Blood tubes were centrifugated at 1500×*g* for 10 min. Then, plasma samples were stored at – 80 °C until analysis.

**Table 1:** Age, sex and body mass index of volunteers selected in the two groups

	Obese adults (*n* = 20)	Normal weight adults (*n* = 20)	*p*-value
Sex*	Women 60%	Women 60%	1
	Men 40%	Men 40%	
Age (years)**	44.9 ± 11.6	30.8 ± 9.5	0.0008
BMI (kg m^−2^)**	40.5 ± 8.6	21.7 ± 2.2	< 0.0001

^*^Statistical analysis by Chi-squared test

^**^Statistical analysis by Mann–Whitney–Wilcoxon test

### Instruments and apparatus

Quantitative analysis was carried out with a 1200 series LC system from Agilent Technologies coupled to an Agilent 6460 triple quadrupole (QqQ) mass spectrometer furnished with an electrospray (ESI) ionization source. Agilent MassHunter Workstation (B.03.01 version, Agilent Technologies, Santa Clara, CA, USA) was the software for data acquisition. Confirmatory analysis was completed with an Agilent 6540 high resolution hybrid quadrupole–time of flight (QTOF) mass spectrometer.

A Midas autosampler with a 100 μL sample loop and a Prospekt-2 system from Spark Holland (Emmen, The Netherlands) were used for sample preparation. The Prospekt-2 system was endowed with a unit for automatic cartridge exchange (ACE) and a high-pressure syringe dispenser (HPD) to aspirate the solutions involved in the sample preparation process. HySphere SPE cartridges from Spark-Holland (10 mm length and 2 mm diameter) packed with polymeric strong anion exchanger (SAX, particle size 25–35 μm) were used to test the retention/elution performance.

### Qualitative analysis of acylcarnitines by LC–MS/MS in DIA mode

Qualitative analysis contributed to confirm the precursor and main product ions selected for MRM transitions. For this purpose, a serum aliquot with physiological levels of ACs was analyzed by the LC–QTOF MS/MS in DIA mode. The injection volume was 10 µL, and the mobile phases and ESI source conditions were those planned for the QqQ. Complementarily, the Q1, skimmer, and octapole voltages were fixed at 130, 65, and 750 V, respectively. The data were acquired in centroid mode in the extended dynamic range (2 GHz) and in All Ions MS/MS mode. Concretely, full scan was carried out at 2.6 spectra s^−1^ within the m/z range of 50–520, using eight collision energy values (0, 15, 20, 22, 25, 28, 30 and 35 eV), and with mass accuracy assured by continuous calibration according to the instructions of the manufacturer.

### Fully automated SPE–LC–MS/MS method for determination of acylcarnitines in human serum

A serum aliquot was thawed in ice to avoid a potential degradation of the analytes owing to a thermal shock. A volume of 20 μL serum was introduced in an opaque vial with a glass insert and diluted with 175 µL of 5 mM pH 9.0 ammonium formate and 5 µL of the IL-ISs multistandard solution at 400 ng mL^–1^. The sequence of automated operations is initiated by solvation of the SPE sorbent cartridge with 2 mL ACN and flow rate 2 mL min^–1^. Then, equilibration of the sorbent was performed with 2 mL of the same buffer solution (5 mM pH 9.0 ammonium formate) at 0.5 mL min^–1^. The sample solution (100 µL) was injected in the system using 0.4 mL of buffer at a flow rate of 0.1 mL min^–1^. The sorbent was washed with 0.4 mL Milli-Q water at 0.1 mL min^–1^. The valve was switched, and the retained compounds were eluted with the mobile phase for 5 min. The valve was switched back to the initial position and cartridge was purged in five steps (2 mL) with water, 1% (v/v) FA, ACN, 1% (v/v) FA and, finally, water at 5 mL min^–1^.

Chromatographic separation of ACs was carried out by an InfinityLab Poroshell 120 EC-C18 analytical column (particle size 2.7 µm, 10 cm length and 2.1 mm inner diameter) from Agilent Technologies, equipped with an InfinityLab Poroshell 120 EC-C18 column guard (particle size 2.7 µm, 5 mm length and 2.1 mm inner diameter) to preserve the integrity of the analytical column. The mobile phases were composed by: (A) 5% (v/v) ACN, 0.1% (v/v) FA and 7.5 mM of ammonium formate in milli-Q water; and (B) 0.1% (v/v) FA in ACN/MeOH/milli-Q water (70:25:5). The initial chromatographic mobile phase was composed of 0% B. A first gradient was applied for 1 min up to 60% mobile phase B, and a second gradient for 4 min up to 80% B, followed by a third gradient for 3 min to 100% B. Then, the final chromatographic mobile phase was kept for 12 min. The temperature of the chromatographic column was set at 30 °C, the flow rate was 300 μL min^−1^ and the time to restore the initial chromatographic conditions was 15 min.

MS/MS detection was carried out in multiple reaction monitoring (MRM) mode in one single run by ESI in positive mode. The capillary voltage was set at + 3.5 kV and the nozzle voltage at 0.5 kV. The nebulizer pressure was 45 psi and the flow and temperature of N_2_ as drying gas 10 L min^–1^ and 325 °C.

Quantitation was carried out by integrating the peak area for each considered AC. The relative amount of each analyte was expressed as percentage of total peak area considering all ACs. Relative contents were also expressed in relative terms by considering calibration models prepared with structurally similar IL-ISs.

## Results and discussion

### Combination of DDA and DIA for screening of acylcarnitines

Metabolomic analysis by LC–MS/MS is generally carried out by DDA or DIA. Both DIA and DDA modes acquire a full MS1 scan, followed by one or multiple MS2 scans. DDA mode acquires all precursor ion in a MS1 scan followed by MS2 scans of the top-n precursor ions ranked by their intensities. Precursor ions are isolated in the collision cell for activation, which means that each MS2 scan is inherently linked to a precursor ion, allowing the elucidation of metabolites. However, DDA mode suffers from a limited acquisition coverage of MS2 spectra due to the preferred selection of abundant precursor ions, and from a undefined MS2 spectra quality since MS2 acquisition is not always performed at the apex of chromatogram peak. In DIA mode, all precursor ions in a predefined isolation window (from 5 Da to a full mass range) are isolated to acquire multiplexed MS2 scan, and this step is repeated until the full mass range is covered, so theoretically, DIA mode enables to acquire either MS1 or MS2 scans for all precursor ions. On the other hand, the associations between precursors and product ions are not identified, but with known fragmentation patterns it is possible to search for these associations (R. Wang et al., 2019b).

MS2 fragmentation of ACs was preliminarily evaluated by analysis of the IL-ISs. All species reported the [M + H]^+^ precursor ion, whereas the most sensitive MRM transition was that involving the formation of the product ion at *m/z* 85. This fits the [C_4_H_5_O_2_]^•^ fragment produced by cleavage of the carnitine moiety and loss of the trimethylamine group. According to this fragmentation pattern, serum samples were analyzed by LC–QTOF MS/MS in DIA mode. Tentative precursor ions from C0 to C24 and the predominant product ion at *m/z* 85.0249 were monitored and correlated along the chromatographic run. Furthermore, we also scanned characteristic fragments of ACs according to the length of the hydrocarbon chain and unsaturation degree of the fatty acid, concretely those generated after the loss of the trimethylamine group (Fig. S1). Thus, ACs were confirmed when precursor ions and both product ions for each analyte were matched by retention time and peak shape (Table S1). This strategy, which provides MS1 and MS2 information of all detected ACs, allowed the identification of 47 species.

**Fig. 1:**
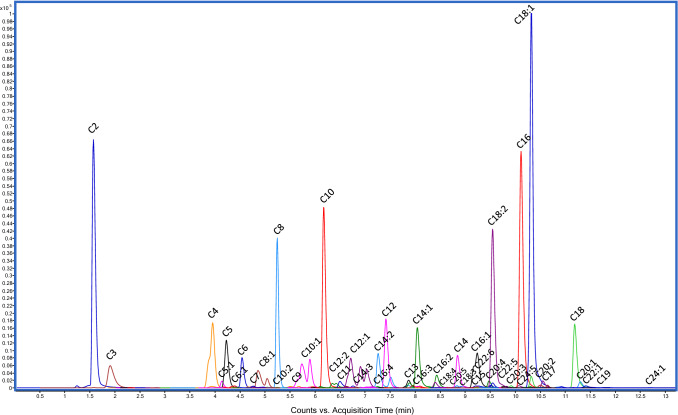
MRM chromatograms of the 47 acylcarnitines detected in the serum pool

After definition of the list of ACs using DIA strategy, the acquisition parameters for DDA MS/MS analysis of ACs with QqQ were optimized. Q1 voltage for filtering precursor ions was tested from 50 to 180 V, while collision energy (CE) for activation of precursor ions was tested from 0 to 40 eV. Figure 1 shows an MRM chromatogram obtained by analysis of the serum pool. Short chain ACs were considered from C2 to C6, which were eluted before min 4.6 retention time. Medium chain ACs included up to C12 and eluted up to min 7.5. Finally, LCACs encompassed from C13 to C24 and eluted from 7 to 12 min. When monounsaturated and polyunsaturated ACs were analogously detected for a specific number of carbons, elution order was carried out by decreasing the number of unsaturated bonds, as shows Table 2 for C14, C16, C18, C20 and C22 ACs (Fig. S2). Concerning the MRM parameters, the Q1 voltage was around 135 V, except for some LCACs such as C19, C22:1, C24:5 and C24:1 that required 150 V. The same trend was observed for the collision energy as this parameter was 15–20 eV for SCACs, 20–25 eV for MCACs, and 25–30 eV for LCACs (Fig. S3). It is worth noting that deuterated IL-ISs were better activated when the CE was decreased up to 17 eV for SCACs and increased with the length of the hydrocarbon chain. Table 2 includes the list of identified ACs in human serum with the optimum LC-QqQ parameters for each one.

**Table 2:** Multiple reaction monitoring parameters for determination of acylcarnitines and isotopically labelled acylcarnitines used as internal standards

Analyte	Analyte abbreviation	Retention time (min)	Quantitation transition	Filtration voltage (V)	Collision energy (eV)	Isotopically labelled acylcarnitines used as internal standards
Acetyl-L-carnitine	C2	1.57	204.2 → 85.0	135	20	C2-d_3_
Acetyl-L-carnitine-d_3_	C2-d_3_	1.57	207.1 → 85.0	80	17	–
Propionyl-L-carnitine	C3	1.91	218.2 → 85.0	135	20	C2-d_3_
Butyryl-L-carnitine	C4	3.95	232.2 → 85.0	135	20	C4-d_3_
Butyryl-L-carnitine-d_3_	C4-d_3_	3.95	235.2 → 85.0	100	17	–
Pentenoyl-L-carnitine	C5:1	4.14	244.2 → 85.0	135	20	C4-d_3_
Valeryl-L-carnitine	C5	4.23	246.2 → 85.0	135	20	C4-d_3_
Hexenoyl-L-carnitine	C6:1	4.39	258.2 → 85.0	135	20	C6-d_3_
Hexanoyl-L-carnitine	C6	4.54	260.2 → 85.0	135	20	C6-d_3_
Hexanoyl-L-carnitine-d_3_	C6-d_3_	4.54	263.2 → 85.0	120	17	–
Heptanoyl-L-carnitine	C7	4.76	274.3 → 85.0	135	20	C6-d_3_
Octenoyl-L-carnitine	C8:1	4.86	286.3 → 85.0	135	20	C6-d_3_
Octanoyl-L-carnitine	C8	5.25	288.3 → 85.0	135	20	C2-d_3_
Nonanoyl-L-carnitine	C9	5.67	302.3 → 85.0	135	20	C10-d_3_
Decadienyl-L-carnitine	C10:2	5.50	312.3 → 85.0	135	20	C10-d_3_
Decenoyl-L-carnitine	C10:1	5.90	314.3 → 85.0	135	20	C10-d_3_
Decanoyl-L-carnitine	C10	6.17	316.3 → 85.0	135	20	C10-d_3_
Decanoyl-L-carnitine-d_3_	C10-d_3_	6.17	319.3 → 85.0	95	21	–
Undecanoyl-L-carnitine	C11	6.50	330.3 → 85.0	135	20	C12-d_9_
Dodecadienoyl-L-carnitine	C12:2	6.35	340.3 → 85.0	135	20	C12-d_9_
Dodecenoyl-L-carnitine	C12:1	6.71	342.3 → 85.0	135	20	C12-d_9_
Lauroyl-L-carnitine	C12	7.41	344.3 → 85.0	135	20	C12-d_9_
Lauroyl-L-carnitine-d_9_	C12-d_9_	7.41	353.3 → 85.0	100	24	–
Tridecanoyl-L-carnitine	C13	7.87	358.3 → 85.0	135	25	C14-d_9_
Myristolinolenoyl-L-carnitine	C14:3	6.63	366.3 → 85.0	135	25	C14-d_9_
Tetradecadienyl-L-carnitine	C14:2	7.26	368.3 → 85.0	135	25	C14-d_9_
Myristoleoyl-L-carnitine	C14:1	8.05	370.3 → 85.0	135	25	C14-d_9_
Myristoyl-L-carnitine	C14	8.85	372.3 → 85.0	135	25	C14-d_9_
Myristoyl-L-carnitine-d_9_	C14-d_9_	8.85	381.3 → 85.0	100	24	–
Pentadecanoyl-L-carnitine	C15	9.51	386.3 → 85.0	135	25	C16-d_3_
Hexadecatetraenoyl-L-carnitine	C16:4	7.25	392.4 → 85.0	135	25	C14-d_9_
Hexadecatrienoyl-L-carnitine	C16:3	7.95	394.3 → 85.0	135	25	C14-d_9_
Hexadecadienoyl-L-carnitine	C16:2	8.40	396.4 → 85.0	135	25	C16-d_3_
Hexadecenoyl-L-carnitine	C16:1	9.24	398.4 → 85.0	135	25	C16-d_3_
Palmitoyl-L-carnitine	C16	10.12	400.3 → 85.0	135	25	C16-d_3_
Palmitoyl-L-carnitine-d_3_	C16-d_3_	10.12	403.3 → 85.0	160	25	–
Heptadecanoyl-L-carnitine	C17	10.65	414.4 → 85.0	135	25	C16-d_3_
Stearidonyl-L-carnitine	C18:4	8.64	420.4 → 85.0	135	25	C14-d_9_
Linolenyl-L-carnitine	C18:3	8.84	422.4 → 85.0	135	25	C14-d_9_
Linoleoyl-L-carnitine	C18:2	9.55	424.4 → 85.0	135	25	C16-d_3_
Oleoyl-L-carnitine	C18:1	10.32	426.4 → 85.0	135	25	C16-d_3_
Stearoyl-L-carnitine	C18	11.19	428.4 → 85.0	135	25	C16-d_3_
Nonadecanoyl-L-carnitine	C19	11.74	442.4 → 85.0	150	30	C16-d_3_
Eicosapentenoyl-L-carnitine	C20:5	8.81	446.3 → 85.0	135	25	C16-d_3_
Arachidonoyl-L-carnitine	C20:4	9.48	448.3 → 85.0	135	25	C16-d_3_
Eicosatrienoyl-L-carnitine	C20:3	9.91	450.4 → 85.0	135	25	C16-d_3_
Eicosadienoyl-L-carnitine	C20:2	10.55	452.4 → 85.0	135	25	C16-d_3_
Eicosenoyl-L-carnitine	C20:1	11.30	454.4 → 85.0	135	25	C16-d_3_
Cervonyl-L-carnitine	C22:6	9.35	472.3 → 85.0	135	25	C14-d_9_
Clupanodonyl-L-carnitine	C22:5	9.69	474.4 → 85.0	135	25	C16-d_3_
Docosatetraenoyl-L-carnitine	C22:4	10.30	476.4 → 85.0	135	25	C16-d_3_
Docosaenoyl-L-carnitine	C22:1	11.56	482.4 → 85.0	150	30	C16-d_3_
Tetracosapentaenoyl-L-carnitine	C24:5	9.95	502.4 → 85.0	150	30	C16-d_3_
Nervonyl-L-carnitine	C24:1	12.69	510.4 → 85.0	150	30	C16-d_3_

### Optimization of the SPE step for sample preparation

ACs are properly ionized at low pH with formic acid. For this reason, the SPE performance was evaluated with an SAX sorbent formed by a divinyl benzene polymer modified with a tertiary amine group, which interacts at suited pH with the negative charge of the carnitine carbonyl group. In addition to the ionic interaction, the SAX sorbent adds a reversed phase interaction through the hydrophobic core around the benzene moiety and the hydrocarbon chain of ACs. By playing with critical variables such as pH, these two mechanisms can be interchanged as primary or secondary interactions. For this reason, the SPE protocol with SAX was optimized by studying the influence of parameters associated to the three crucial steps: loading, washing and elution (Table S2). Before sample loading, the sorbent was conditioned and equilibrated with 2 mL of 2% ammonia in water (pH 9) at 0.5 mL min^–1^.

Sample loading was optimized by evaluating three parameters associated to the carrier solution: flow rate, volume, and composition. The optimum sample loading was carried out with 0.4 mL water pH 9 adjusted with ammonia at low flow rate, 0.1 mL min^–1^, to maximize interaction of ACs with the sorbent. Complementarily, ACN was omitted in this step to avoid elution of carnitine and SCACs. For loading, serum (20 µL) was diluted with 5 µL of the IL-ISs multistandard solution at 400 ng mL^–1^ and 175 µL of the selected loading solution to ensure deprotonation of the carboxyl groups, thus favoring anionic interactions with the sorbent.

The inclusion of a washing step was valued to improve the ionization efficiency. This step was also carried out at 0.1 mL min^–1^ to avoid partial elution of ACs with 0.4 mL pH 7 water. In this step, ACN did not lead to partial elution of polar carnitines, but it did not improve the analytical response in MS.

**Fig. 2:**
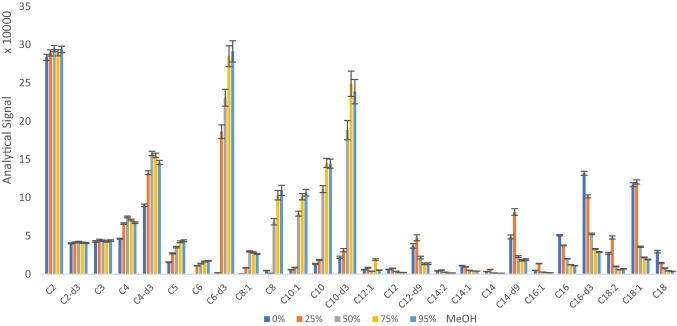
Influence of the organic mobile phase composition on the ionization of the predominant acylcarnitines, represented by MeOH concentration in phase B

Concerning elution, we evaluated the influence of the organic solvent in the mobile phase and the elution time. Comparison between ACN and MeOH revealed differences in the ionization efficiency. Thus, an ionization attenuation effect was detected in MCACs with ACN, whereas ionization suppression effects affected to LCACs with MeOH, especially to predominant C16 and C18 ACs (Figs. 2 and S4). A compromise solution was found with ACN/MeOH/water (70:25:5) to favor the ionization of MCACs and LCACs. An increase of ACN concentration promoted coelution of MCACs with other polar lipids such as glycerophospholipids. On the other hand, MeOH delayed the elution of these ionization suppressors affecting specially to LCACs. Finally, the elution time was optimized considering the time the valve is switch and the mobile phase is going through the cartridge. The switch valve was activated for 5 min to guarantee a quantitative elution of the target analytes. Longer elution times led to a decrease of sensitivity by coelution of interferents.

The extraction efficiency was evaluated with the resulting SPE protocol considering the wide polarity range of the analytes, from low polar carnitine and SCACs to LCACs. For this purpose, a double cartridge configuration was used to evaluate the retention/elution capacity of the sorbent. This configuration allows passing the injected sample through two on-line connected cartridges and the retained material is sequentially eluted to the LC–MS/MS system by switching a selection valve of each cartridge. The extraction efficiency was measured by comparing the content of each AC retained in the two cartridges by using the following formula:

$$\% Efficiency = \frac{P1}{{P1 + P2}} \times 100$$

being P1 and P2 the quantitative responses of the analytes retained in the cartridges 1 and 2, respectively. According to this formula, Table 3 shows the extraction efficiency expressed as percentage with standard deviation. Extraction was quantitative for most ACs except for polar SCACs C2, C3 and C4, and for less polar LCACs C18, C19, C20:1, C22:1 and C24:1. This result could be explained by an incomplete retention in the sorbent, since the extraction efficiency ranged between 35.1–51.4% and 61.5–83.3%, respectively. However, variability levels were particularly low, even for the most polar compounds and, in addition, IL-ISs reported a similar performance in the SPE process.

**Table 3:** Extraction efficiency and variability, expressed within-day and between-days, for each acylcarnitine

Analyte	Extraction efficiency ± SD (%)	Within-day variability (%)	Between-days variability (%)
C2	35.45 ± 1.05	2.8	14.6
C2-d_3_	35.09 ± 1.20		
C3	38.24 ± 1.98	4.5	11.5
C4	51.35 ± 1.07	3.0	10.3
C4-d_3_	53.14 ± 1.06		
C5:1	87.17 ± 0.12	1.9	7.5
C5	92.07 ± 0.41	5.7	5.9
C6:1	99.52 ± 0.58	4.8	15.6
C6	99.93 ± 0.08	6.2	14.2
C6-d_3_	99.99 ± 0.01		
C7	99.58 ± 0.01	4.6	9.1
C8:1	99.97 ± 0.01	5.3	9.7
C8	99.99 ± 0.01	12.0	18.9
C9	99.68 ± 0.20	9.2	12.6
C10:2	99.11 ± 0.39	3.6	5.5
C10:1	100.00 ± 0.00	8.2	10.8
C10	99.99 ± 0.00	8.2	11.5
C10-d_3_	99.99 ± 0.00		
C11	99.90 ± 0.12	14.2	17.0
C12:2	99.92 ± 0.09	12.4	18.0
C12:1	99.98 ± 0.02	11.7	17.1
C12	99.80 ± 0.05	10.9	14.9
C12-d_9_	99.95 ± 0.00		
C13	99.96 ± 0.01	5.5	11.2
C14:3	99.96 ± 0.02	10.8	17.0
C14:2	100.00 ± 0.00	11.6	15.1
C14:1	99.96 ± 0.02	5.6	12.4
C14	99.54 ± 0.09	5.4	12.9
C14-d_9_	99.92 ± 0.01		
C15	99.97 ± 0.00	4.0	6.1
C16:4	99.97 ± 0.01	7.6	13.1
C16:3	99.97 ± 0.02	6.1	13.2
C16:2	99.78 ± 0.08	6.7	6.8
C16:1	99.93 ± 0.04	4.3	8.7
C16	98.85 ± 0.16	2.9	11.1
C16-d_3_	98.84 ± 0.23		
C17	88.10 ± 0.42	8.5	9.8
C18:4	98.59 ± 1.05	2.6	20.3
C18:3	99.93 ± 0.00	6.1	10.4
C18:2	99.92 ± 0.02	8.5	14.0
C18:1	97.13 ± 0.31	4.6	19.3
C18	69.81 ± 1.22	11.8	10.5
C19	61.95 ± 0.88	14.5	12.5
C20:5	99.21 ± 0.61	3.9	10.0
C20:4	99.98 ± 0.01	3.9	6.4
C20:3	99.88 ± 0.02	5.4	19.6
C20:2	90.13 ± 1.36	12.9	17.3
C20:1	68.83 ± 2.85	10.4	13.0
C22:6	98.55 ± 1.51	7.9	11.3
C22:5	98.85 ± 0.48	7.1	15.6
C22:4	95.80 ± 0.21	7.6	19.2
C22:1	61.50 ± 17.06	11.2	18.4
C24:5	93.05 ± 8.14	5.0	19.6
C24:1	83.25 ± 5.13	9.8	10.9

Values were estimated in percentage and measured at physiological levels using a serum pool

To test the reusability of the sorbent cartridge 10 serum aliquots were analyzed in a sequence. The relative concentration for each analyte was plotted in the different aliquots considering confidence intervals with significance level (*α*) 0.01 (Fig. S5). With this strategy, reusability was set at 5 analyses conditioned by C20:2 and C24:5 ACs.

**Fig. 3:**
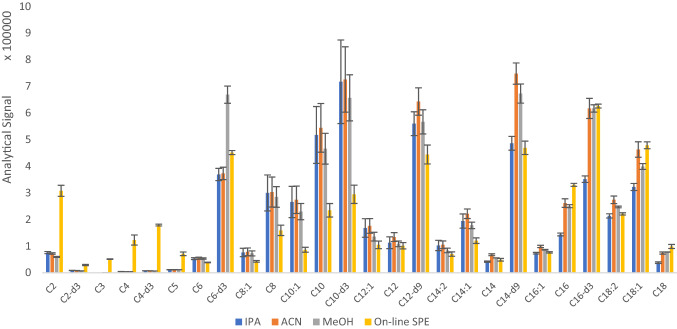
Comparison of the quantitative signal for predominant acylcarnitines provided by the proposed method and protein precipitation using different organic solvents (IPA, MeOH and ACN)

The SPE performance was compared with a conventional sample preparation process based on protein precipitation by testing common solvents used for this task: IPA, ACN and MeOH. To evaluate interferents removal and detection response, protein precipitation was carried out by adding 600 µL of organic solvent to 200 µL serum aliquots. The supernatants were isolated, preconcentrated by evaporation to dryness and reconstituted in 200 µL of ACN/water (90:10). To compare the performance with automated SPE, we injected 10 µL of the reconstituted solution. As Fig. 3 (Fig. S6 for low concentrated ACs) shows, the SPE clean-up was especially efficient for SCACs and LCACs by reducing the influence of highly polar and non-polar interferents. However, coeluting species with ionization competitivity affected to C6–C12 MCACs in SPE, and protein precipitation resulted in a best detection capability.

### Characterization of the method

The method was evaluated in terms of calibration linearity, sensitivity, accuracy and precision. Linearity was estimated by preparing calibration models with the set of IL-ISs spiked to serum aliquots in a wide range of concentrations ranging from 1 pg mL^–1^ to 50 ng mL^–1^ (Table 4). Determination coefficients were in all cases above 0.991. Limits of quantitation (LOQs) were calculated as the lowest levels of the calibration ranges considering a maximum deviation of ± 20% for inclusion. LOQs ranged from 1 pg mL^–1^ for SCACs and LCACs to 2.5–10 pg mL^–1^ for C10-d_3_ and C6-d_3_, respectively. These two MCACs were among species affected by ionization suppression effects. Limits of detection (LODs) were estimated as the concentration that provides the three-times signal above the background noise and confirmed by analysis of serum aliquots spiked at the lowest concentration levels of each calibration range.

**Table 4:** Characterization of the SPE–LC–MS/MS method for determination of acylcarnitines

Analyte	Linear range (ng mL^–1^)	Determination coefficient (*R*^2^)	Calibration equation	LOD (pg mL^–1^)	LOQ (pg mL^–1^)	Accuracy (%)*			
						10 ng** mL**^**–1**^	25 ng** mL**^**–1**^	50 ng** mL**^**–1**^	100 ng** mL**^**–1**^
C2-d_3_	0.001–50	0.994	y = 7.0056x − 1903.6	0.33	1	105.4	108.7	114.8	75.7
C4-d_3_	0.001–50	0.998	y = 55.324x − 4923	0.33	1	106.8	94.5	109.0	111.0
C6-d_3_	0.01–10	0.998	y = 213.54x − 1151	3.33	10	112.8	90.5	95.6	101.6
C10-d_3_	0.0025–10	0.991	y = 150.04x − 13570	0.75	2.5	113.2	80.7	88.6	103.9
C12-d_9_	0.001–10	0.993	y = 274.46x − 24614	0.33	1	107.2	84.5	89.8	103.4
C14-d_9_	0.001–10	0.997	y = 250.23x − 9085.6	0.33	1	106.1	88.0	93.1	102.4
C16-d_3_	0.001–10	0.999	y = 246.74x − 8332.8	0.33	1	111.5	100.4	100.9	100.9

^*^Concentration in serum

The accuracy of the method was evaluated by triplicate analysis of serum samples spiked with the IL-ISs at four concentration levels, 10, 25, 50 and 100 ng mL^–1^, which represent a range of typical concentrations for ACs in serum (Psychogios et al., 2011; Trabado et al., 2017; M. Wang et al., 2019a). Accuracy levels were within ± 25% at the four concentration levels and, therefore, no trends were detected by polarity or concentration (Table 4). In fact, accuracy was properly estimated for SCACs despite they reported a low extraction efficiency in the SPE process.

To evaluate precision, four serum aliquots were analyzed in duplicates for three consecutive days to evaluate the within-day and between-days variability expressed as relative standard deviation (RSD). Variability was calculated for all ACs by using concentrations expressed in relative terms as percentage of total peak area considering all ACs. Within-day variability did not exceed 15% of variability, whereas the between-days variability was always below 20% (Table 3). According to these values, precision of the method is adequate for ACs profiling.

Table 5 compares the proposed method with other reported approaches according to the operational and analytical features. Thus, the proposed method is fully automated replacing protein precipitation by on-line SPE. In addition, no derivatization is required. Precision of the method developed here was at intermediate values, but the number of detected species was clearly above to those included in other methods dealing with determination of ACs.

**Table 5:** Comparison of the proposed method with other methods reported in the literature for determination of lineal ACs

Method	Sample volume (µL)	Fully automation	Protein precipitation	Solid phase extraction	Derivatization	Chromatographic mode	Between-days variability	Number of lineal ACs measured
Proposed method	20	Yes	No	SAX	No	RP-LC	< 20.3%	47
Giesbertz et al., 2015	10	No	MeOH	No	Butyl esters	RP-LC	< 38.7%	24
Gucciardi et al., 2012	6	No	MeOH	No	Butyl esters	RP-LC	< 15.2%	22
Peng et al., 2013	20	No	ACN	No	No	HILIC	< 16.6%	21

### Application of the developed method to determine physiological levels of the target analytes in human serum

A methodological application study was carried out by analysis of serum samples from forty volunteers (20 obese adults and 20 normal weight adults). Serum aliquots were analyzed using the developed method to obtain the ACs profile. Table 6 lists relative concentrations expressed as percentage. Predominant ACs were C10, C16, C18:1 and C18:2, which surpassed 5% relative concentration. According to this profile, LCACs were the most concentrated forms (around 60%), followed by MCACs (around 30%) and, finally, SCACs (around 10%). Similar concentrations were found for saturated and monounsaturated ACs (around 42%), while polyunsaturated ACs were found at lower concentrations (around 14%). Statistical analysis allowed finding significant differences by Mann–Whitney–Wilcoxon test in the relative concentration of some ACs by comparing profiles of normal weight and obese individuals. These were C7 (*p*-value 0.006), C8 (< 0.0001), C10 (< 0.0001), C16 (< 0.0001), C19 (0.0005), C20:1 (0.0009), and C22:4 (0.008). Significant differences were also found in the content of LCACs (0.004). Significant MCACs C7, C8 and C10 were found at higher concentrations in normal weight subjects as compared to obese individuals, while the opposite result was found for LCACs C16, C20:1 and C22:4 that were significantly higher in obese volunteers. As expected, the same trend was found for the LCACs sum. On the other hand, the low concentrated LCAC C19 was found at higher level in normal weight individuals (Fig. S7).

**Table 6:** Relative concentrations of acylcarnitines expressed as percentage for the two groups of individuals evaluated in this study

Analyte	Obese adults (n = 20)	Normal weight adults (n = 20)	*p*-value
	Mean ± SD		
C2	2.81 ± 0.78	3.42 ± 1.08	
C3	0.50 ± 0.22	0.49 ± 0.18	
C4	1.53 ± 0.67	1.55 ± 0.56	
C5:1	0.22 ± 0.11	0.27 ± 0.10	
C5	3.20 ± 1.47	3.31 ± 1.28	
C6:1	0.05 ± 0.03	0.05 ± 0.02	
C6	0.88 ± 0.26	1.05 ± 0.28	
C7	0.09 ± 0.05	0.11 ± 0.04	0.0061
C8:1	1.54 ± 0.72	1.09 ± 0.59	
C8	3.93 ± 0.79	6.49 ± 2.04	< 0.0001
C9	0.23 ± 0.14	0.22 ± 0.09	
C10:2	0.23 ± 0.09	0.23 ± 0.16	
C10:1	4.67 ± 1.72	4.46 ± 1.42	
C10	7.34 ± 2.06	10.60 ± 2.88	0.0007
C11	0.61 ± 0.51	0.68 ± 0.31	
C12:2	0.58 ± 0.21	0.51 ± 0.16	
C12:1	4.70 ± 1.20	4.64 ± 0.98	
C12	3.58 ± 1.41	3.47 ± 0.74	
C13	0.52 ± 0.20	0.82 ± 0.59	
C14:3	0.20 ± 0.06	0.24 ± 0.07	
C14:2	2.21 ± 0.52	2.21 ± 0.67	
C14:1	4.72 ± 0.95	5.07 ± 1.03	
C14	2.79 ± 0.84	2.18 ± 0.42	
C15	0.45 ± 0.21	0.52 ± 0.19	
C16:4	0.13 ± 0.05	0.12 ± 0.04	
C16:3	0.27 ± 0.06	0.24 ± 0.06	
C16:2	0.56 ± 0.10	0.53 ± 0.12	
C16:1	3.66 ± 0.86	3.30 ± 0.68	
C16	9.82 ± 2.44	5.50 ± 1.92	< 0.0001
C17	0.39 ± 0.10	0.50 ± 0.17	
C18:4	0.03 ± 0.009	0.03 ± 0.009	
C18:3	0.25 ± 0.05	0.24 ± 0.06	
C18:2	8.57 ± 1.91	7.99 ± 2.90	
C18:1	24.42 ± 5.92	23.41 ± 5.69	
C18	1.94 ± 0.43	2.36 ± 0.60	
C19	0.02 ± 0.003	0.02 ± 0.006	0.0005
C20:5	0.03 ± 0.01	0.04 ± 0.01	
C20:4	0.33 ± 0.11	0.32 ± 0.14	
C20:3	0.32 ± 0.11	0.25 ± 0.09	
C20:2	0.36 ± 0.15	0.30 ± 0.09	
C20:1	0.55 ± 0.13	0.45 ± 0.08	0.0009
C22:6	0.05 ± 0.02	0.06 ± 0.02	
C22:5	0.02 ± 0.008	0.02 ± 0.009	
C22:4	0.05 ± 0.02	0.04 ± 0.02	0.008
C22:1	0.02 ± 0.007	0.02 ± 0.005	
C24:5	0.02 ± 0.005	0.02 ± 0.004	
C24:1	0.10 ± 0.04	0.12 ± 0.04	
SCACs	9.25 ± 2.56	10.19 ± 2.01	
MCACs	27.62 ± 6.05	32.65 ± 6.60	
LCACs	63.13 ± 5.89	57.16 ± 6.74	0.0043
Saturated ACs	40.84 ± 4.87	43.51 ± 4.88	
Monounsaturated ACs	44.86 ± 5.14	43.07 ± 4.69	
Polyunsaturated ACs	14.30 ± 2.25	13.42 ± 3.57	

These results agreed to those reported in previous studies involving no diabetic and type-2 diabetic obese patients and normal weight individuals. These two obese groups showed elevated LCAC levels, which can be explained by an impaired β-oxidation and mitochondrial dysfunction (Mihalik et al., 2010). On the other hand, Bene et al. (2013) reported that metabolic syndrome and type-2 diabetes obese patients provided low MCAC and LCAC levels as compared to the control group. Therefore, LCAC results contrasted to those obtained in our study, which seems to be contradictory since obesity induces an accumulation of ACs owing to β-oxidation. Manta-Vogli et al. (2021) recently reported elevated LCAC levels and low MCAC contents in a high weight (> 3500 g) newborn group, with similar results to those found in our study. This suggests a variability in ACs profile associated to body weight regardless of the age.

Obesity is a well-known risk factor for diabetes development and Mai et al. (2013) compared ACs levels between individuals at different predibetic states. Elevated levels of LCACs (C18:1) and MCACs (C14:1, C14:2) were found in overweight patients with impaired glucose tolerance, which suggest that high AC levels cannot be exclusively explained by obesity. Another study carried out by Guasch-Ferré et al. (2019) found that SCAC and LCAC levels were significantly associated with high cardiovascular risk in a Mediterranean cohort. Zheng et al. (2021) related the high levels of LCACs and MCACs with risk of diabetic cardiomyopathy in non-obese patients.

Concerning physiological contents, relative concentrations were estimated by equivalence to IL-ISs (Table S3). Similar levels of ACs were found in the two groups of individuals, which were close to those reported by authors in adults for common ACs, except for C8:1 and C18:1 that gave some differences. It is worth mentioning that C2 acetyl-L-carnitine was the most concentrated ACs found in human serum. However, this result does not fit the relative content expressed as percentage due to the low sensitivity in the quantitative response. Similarly, C3 and C4 were also affected by low sensitivity in the estimation expressed as percentage. With these premises, quantitation using calibration models should be used to monitor the performance of individual ACs, while the relative profile is useful to compare groups of individuals.

## Conclusion

An overall strategy was developed for identification, confirmatory analysis and quantitation of ACs in human serum. The fragmentation pattern allowed configuring a DIA method for identification and confirmation of 47 ACs, classified by the hydrocarbon length and the number of unsaturations. For quantitative analysis, a DDA method based on SPE–LC–MS/MS was optimized to achieve high sensitivity and selectivity as compared to the conventional protein precipitation. This fact allows simplifying the sample preparation step to serum dilution, using a low sample volume. The combination of DIA and DDA-MRM methods in LC–MS/MS supports the identification and quantitation of numerous analogous metabolites characterized by a common fragmentation scheme. The proposed strategy has been applied to a cohort of normal weight and obese individuals to determine reference levels as percentage and concentrations.

## Supplementary Information

Below is the link to the electronic supplementary material.

Supplementary file1 (PDF 1698 kb)
