# Supplementary material for: Combining data acquisition modes in liquid-chromatography–tandem mass spectrometry for comprehensive determination of acylcarnitines in human serum
Source: Metabolomics. 2022 Jul 21;18(8):59. doi: 10.1007/s11306-022-01916-5 (PMC9300566; doi:10.1007/s11306-022-01916-5)
Supplement: Supplementary file 1 — Supplementary file1 (PDF 1698 kb) [file 11306_2022_1916_MOESM1_ESM.pdf]

Electronic supplementary information

Combining data acquisition modes in liquid-chromatography–tandem mass spectrometry for comprehensive determination of acylcarnitines in human serum

D. Luque-Córdoba[a,b,c,d], M. Calderón-Santiago[a,b,c,d] and F. Priego-Capote*[a,b,c,d]

[a]Department of Analytical Chemistry, Annex Marie Curie Building, Campus of Rabanales, University of Córdoba, Córdoba, Spain.

[b]Nanochemistry University Institute (IUNAN), Campus of Rabanales, University of Córdoba, Córdoba, Spain.

[c]Maimónides Institute of Biomedical Research (IMIBIC), Reina Sofía University Hospital, University of Córdoba, Córdoba, Spain.

[d]Consortium for Biomedical Research in Frailty & Healthy Ageing, CIBERFES, Carlos III Institute of Health, Spain.

*Corresponding author: Phone: +34 957 218615, e-mail: feliciano.priego@uco.es (F. Priego-Capote).

E-mail: feliciano.priego@uco.es (F. Priego-Capote)

Table S1. Parameters for confirmatory analysis of 47 acylcarnitines by LC–QTOF MS/MS in DIA mode.

Abbreviation	Retention time (min)	Precursor ion	Common product ion	Characteristic product ion	Collision energy (eV)	LIPID MAPS ID	HMDB ID
C2	1.217	204.1230	85.0249	145.0495	22	LMFA07070050	HMDB0000201
C2-d_3	1.217	207.1419	85.0249	148.0684	22	-	
C3	1.217	218.1387	85.0249	159.0652	22	LMFA07070005	HMDB0000824
C4	4.557	232.1543	85.0249	173.0808	20	LMFA07070003	HMDB0002013
C4-d_3	4.557	235.1732	85.0249	173.0808	20	-	
C5:1	4.624	244.1543	85.0249	185.0808	20		
C5	4.758	246.1700	85.0249	187.0965	22	LMFA07070076	HMDB0013128
C6:1	4.741	258.1700	85.0249	199.0965	22	LMFA07070031	HMDB0013161
C6	4.942	260.1856	85.0249	201.1121	28	LMFA07070070	HMDB0000756
C6-d_3	4.958	263.2045	85.0249	204.1310	25	-	
C7	5.175	274.2013	85.0249	215.1278	22	LMFA07070068	HMDB0013238
C8:1	5.259	286.2013	85.0249	227.1278	25	LMFA07070014	HMDB0240723
C8	5.610	288.2169	85.0249	229.1434	25	LMFA07070095	HMDB0000791
C9	5.677	302.2326	85.0249	243.1591	25	LMFA07070082	HMDB0013288
C10:2	5.677	312.2169	85.0249	253.1434	22	LMFA07070015	HMDB0250913
C10:1	6.011	314.2326	85.0249	255.1591	28	LMFA07070017	HMDB0240772

C10	6.295	316.2482	85.0249	257.1747	28	LMFA07070059	HMDB0000651
C10-d$_3$	6.278	319.2671	85.0249	260.1936	25	-	
C11	6.545	330.2639	85.0249	271.1904	28	LMFA07070110	HMDB0013321
C12:2	6.261	340.2482	85.0249	281.1747	28	ChEBI ID CHEBI:85447	
C12:1	6.829	342.2639	85.0249	283.1904	28	ChEBI ID CHEBI:85446	
C12	7.280	344.2795	85.0249	285.2060	28	LMFA07070062	HMDB0002250
C12-d$_9$	7.280	353.3360	85.0249	285.2060	25	-	
C13	7.631	358.2952	85.0249	299.2217	30		HMDB0241308
C14:3	6.679	366.2639	85.0249	307.1904	28		
C14:2	7.146	368.2795	85.0249	309.2060	28	LMFA07070020	HMDB0258883
C14:1	7.748	370.2952	85.0249	311.2217	25	LMFA07070057	HMDB0240588
C14	8.483	372.3108	85.0249	313.2373	28	LMFA07070102	HMDB0005066
C14-d$_9$	8.483	381.3673	85.0249	313.2373	25	-	
C15	8.934	386.2365	85.0249	327.1630	30		HMDB0062517
C16:4	7.112	392.2795	85.0249	333.2060	28	BiGG ID hd691215crn	
C16:3	7.748	394.2956	85.0249	335.2221	28	BiGG ID hd6912crn_c	
C16:2	8.082	396.3108	85.0249	337.2373	28	LMFA07070021	HMDB0013334

C16:1	8.934	398.3265	85.0249	339.2530	28	LMFA07070097	HMDB0006317
C16	9.836	400.3421	85.0249	341.2686	28	LMFA07070079	HMDB0000222
C16-d_3	9.836	403.3610	85.0249	341.2686	25	-	
C17	10.353	414.3578	85.0249	355.2843	28	LMFA07070067	HMDB0006210
C18:4	8.298	420.3108	85.0249	361.2373	25	LMFA07070106	HMDB0006463
C18:3	8.550	422.3265	85.0249	363.2530	28	LMFA07070112	HMDB0006318
C18:2	9.284	424.3421	85.0249	365.2686	30	LMFA07070092	HMDB0006469
C18:1	10.086	426.3578	85.0249	367.2843	30	LMFA07070096	HMDB0005065
C18	10.888	428.3734	85.0249	369.2999	28		HMDB0000848
C19	11.422	442.3891	85.0249	383.3156	35	BiGG ID M02611	
C20:5	8.433	446.3265	85.0249	387.2530	35		
C20:4	9.202	448.3421	85.0249	389.2686	30	LMFA07070088	HMDB0006455
C20:3	9.601	450.3578	85.0249	391.2843	35	BiGG ID M00011	
C20:2	10.185	452.3734	85.0249	393.2999	35	LMFA07070011	HMDB0240747
C20:1	11.004	454.3891	85.0249	395.3156	30	LMFA07070010	HMDB0240746
C22:6	9.066	472.3421	85.0249	413.2686	30	LMFA07070055	HMDB0006510
C22:5	9.400	474.3578	85.0249	415.2843	35	LMFA07070058	HMDB0006496
C22:4	10.068	476.3734	85.0249	417.2999	35	LMFA07070022	HMDB0240758

C22:1	11.253	482.4204	85.0249	423.3469	30		
C24:5	9.668	502.3891	85.0249	443.3156	35		
C24:1	12.206	510.4517	85.0249	451.3782	35		HMDB0006509

Table S2. Optimization of the main variables involved in the three steps of the on-line SPE process.

Step	Variables	Studied range	Selected value	Explanation
**Loading**	Flow rate	0.1–0.5 mL min^{-1}	0.1 mL min^{-1}	Maximizes interaction of ACs with the sorbent.
	Composition	0–10 % ACN pH 8–10	0 % ACN pH 9	Ensures deprotonation of the carboxyl groups, thus favoring anionic interactions with the sorbent.
	Volume	0.6–1.4 mL	0.4 mL	There is not elution of analytes.
**Washing**	Flow rate	0.1–1 mL min^{-1}	0.1 mL min^{-1}	Does not disrupt analyte-sorbent interaction.
	Composition	0–20 % ACN pH 7–9	0 % ACN pH 7	Clean-up of polar interferents.
	Volume	0.6–1.4 mL	0.4 mL	Minimizes elution of analytes.
**Elution**	Chromatographic mobile phase	0–95 % MeOH	25 % MeOH	A compromise solution for the attenuation effect in MCACS and LCACs.
	Time	5–15 min	5 min	Short elution time explained by the significant decrease of pH.

Table S3. Physiological levels of acylcarnitines found in human serum for the two groups of individuals.

Analyte	Obese adults	Normal weight adults	Range reported in the literature (ng mL^{-1})	Reference
	Mean ± SD (ng mL^{-1})			
C2	573.15 ± 161.12	570.25 ± 124.05	2246.7 ± 612.7	Wang et al.,2019
C3	102.85 ± 34.45	86.76 ± 30.86	110.2 – 158.2	Wang et al.,2019
C4	54.01 ± 22.92	45.34 ± 13.49	48.5 ± 20.8	Trabado et al., 2017
C5:1	7.01 ± 3.21	7.51 ± 3.31		
C5	29.8 ± 11.3	27.2 ± 13.2	24 – 35.5	Wang et al.,2019
C6:1	0.46 ± 0.22	0.39 ± 0.13		
C6	8.46 ± 1.60	9.12 ± 3.50	18.6 – 27.9	Wang et al.,2019
C7	0.82 ± 0.48	0.91 ± 0.39		
C8:1	15.21 ± 7.35	9.20 ± 4.63	57.0 ± 42.8	Psychogios et al., 2011
C8	39.38 ± 10.94	59.34 ± 35.59	62.9 – 102.3	Wang et al.,2019
C9	3.10 ± 1.55	2.96 ± 0.83		
C10:2	2.97 ± 0.77	2.91 ± 1.11		
C10:1	43.24 ± 13.11	42.25 ± 16.20	53.2 ± 12.5	Psychogios et al., 2011
C10	68.74 ± 21.79	102.10 ± 53.17	84 – 137.4	Wang et al.,2019
C11	5.16 ± 3.51	6.58 ± 2.62		
C12:2	4.92 ± 1.38	5.06 ± 1.35		
C12:1	34.58 ± 13.05	39.94 ± 13.87		
C12	26.05 ± 10.46	30.43 ± 12.33	23.6 – 39.1	Wang et al.,2019
C13	3.13 ± 1.11	5.65 ± 4.05		
C14:3	2.33 ± 0.41	2.84 ± 0.64		
C14:2	16.67 ± 5.12	19.60 ± 7.75		
C14:1	26.17 ± 8.27	32.69 ± 12.52		
C14	15.59 ± 5.41	14.16 ± 4.22	10 – 14.7	Wang et al.,2019
C15	2.08 ± 1.54	4.64 ± 2.83		
C16:4	1.81 ± 0.31	1.91 ± 0.43		
C16:3	1.86 ± 0.50	1.88 ± 0.52		
C16:2	3.39 ± 0.85	3.71 ± 1.20		

C16:1	19.15 ± 9.01	30.74 ± 14.74		
C16	48.23 ± 12.29	46.00 ± 11.49	56.7 ± 11.1	Wang et al.,2019
C17	1.73 ± 0.83	4.32 ± 2.23		
C18:4	0.55 ± 0.07	0.55 ± 0.06		
C18:3	1.71 ± 0.37	1.89 ± 0.56		
C18:2	45.11 ± 21.01	78.55 ± 48.07	25.4 ± 12.7	Psychogios et al., 2011
C18:1	131.70 ± 68.74	213.52 ± 80.74	107.4 ± 26.4	Wang et al.,2019
C18	9.89 ± 4.31	21.38 ± 8.08	17.8 ± 4.1	Wang et al.,2019
C19	0.01 ± 0.01	0.06 ± 0.03		
C20:5	0.55 ± 0.06	0.58 ± 0.07		
C20:4	1.51 ± 1.07	2.79 ± 2.20		
C20:3	1.42 ± 1.05	1.98 ± 1.29		
C20:2	1.49 ± 0.83	2.49 ± 1.43		
C20:1	2.52 ± 1.12	3.80 ± 1.63		
C22:6	0.07 ± 0.05	0.15 ± 0.08		
C22:5	0.03 ± 0.03	0.05 ± 0.04		
C22:4	0.07 ± 0.05	0.10 ± 0.07		
C22:1	0.01 ± 0.01	0.03 ± 0.03		
C24:5	0.47 ± 0.04	0.49 ± 0.03		
C24:1	0.15 ± 0.07	0.35 ± 0.16		

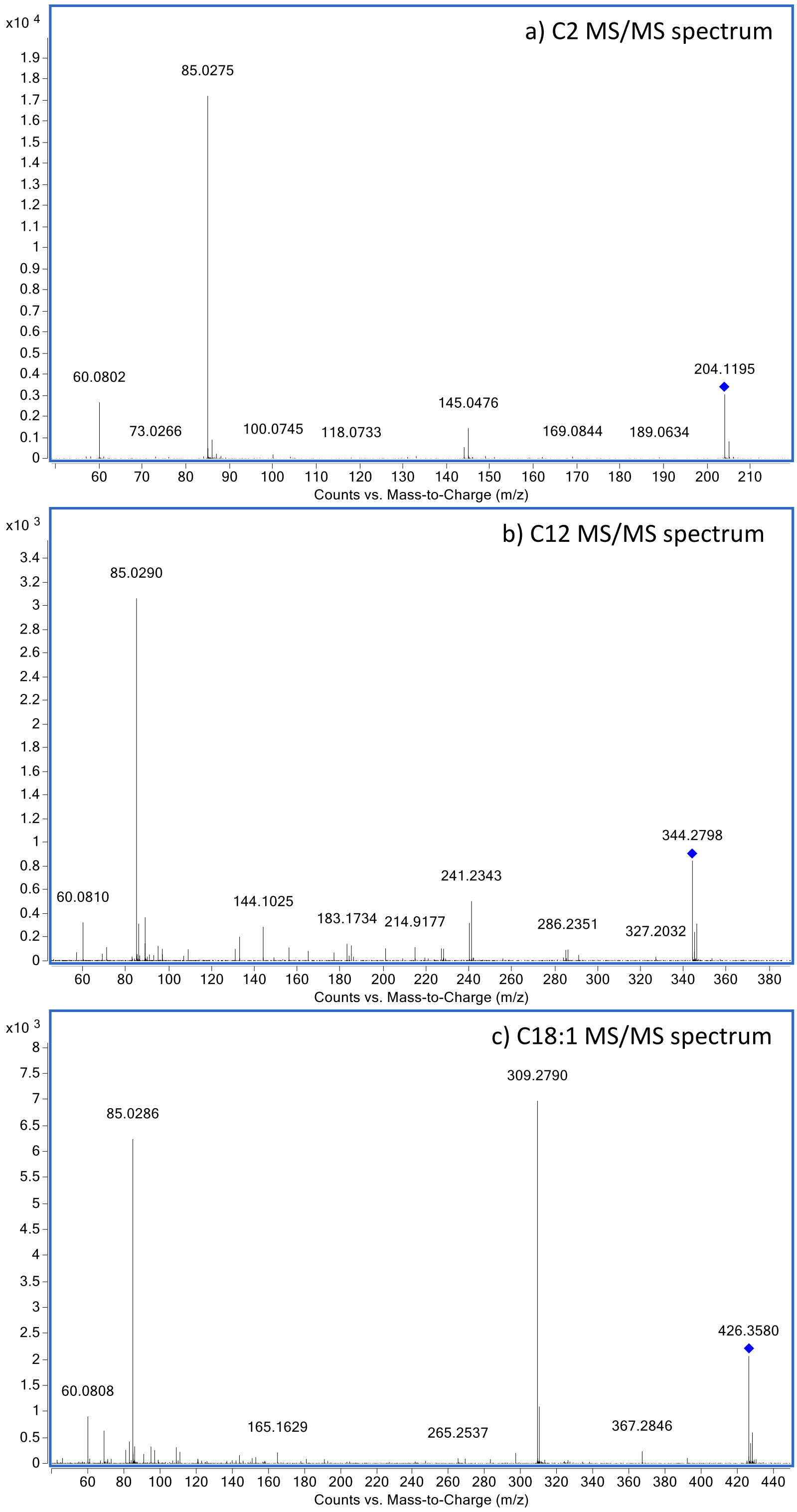

Fig. S1. MS/MS spectrum for a a) SCAC (C2), b) MCAC (C12) and c) LCAC (C18:1).

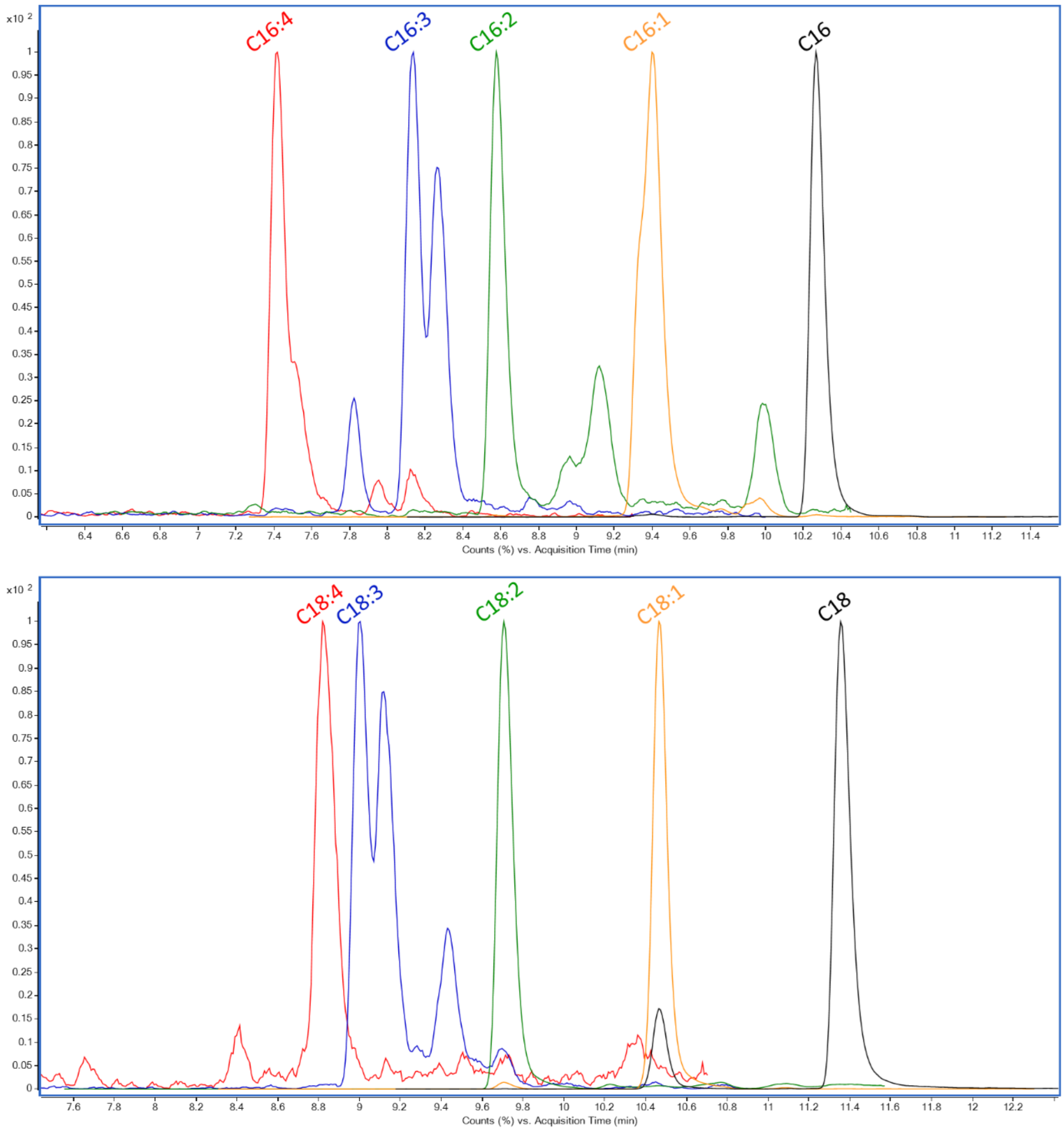

Fig. S2. MRM chromatograms of C16 and C18 acylcarnitine species.

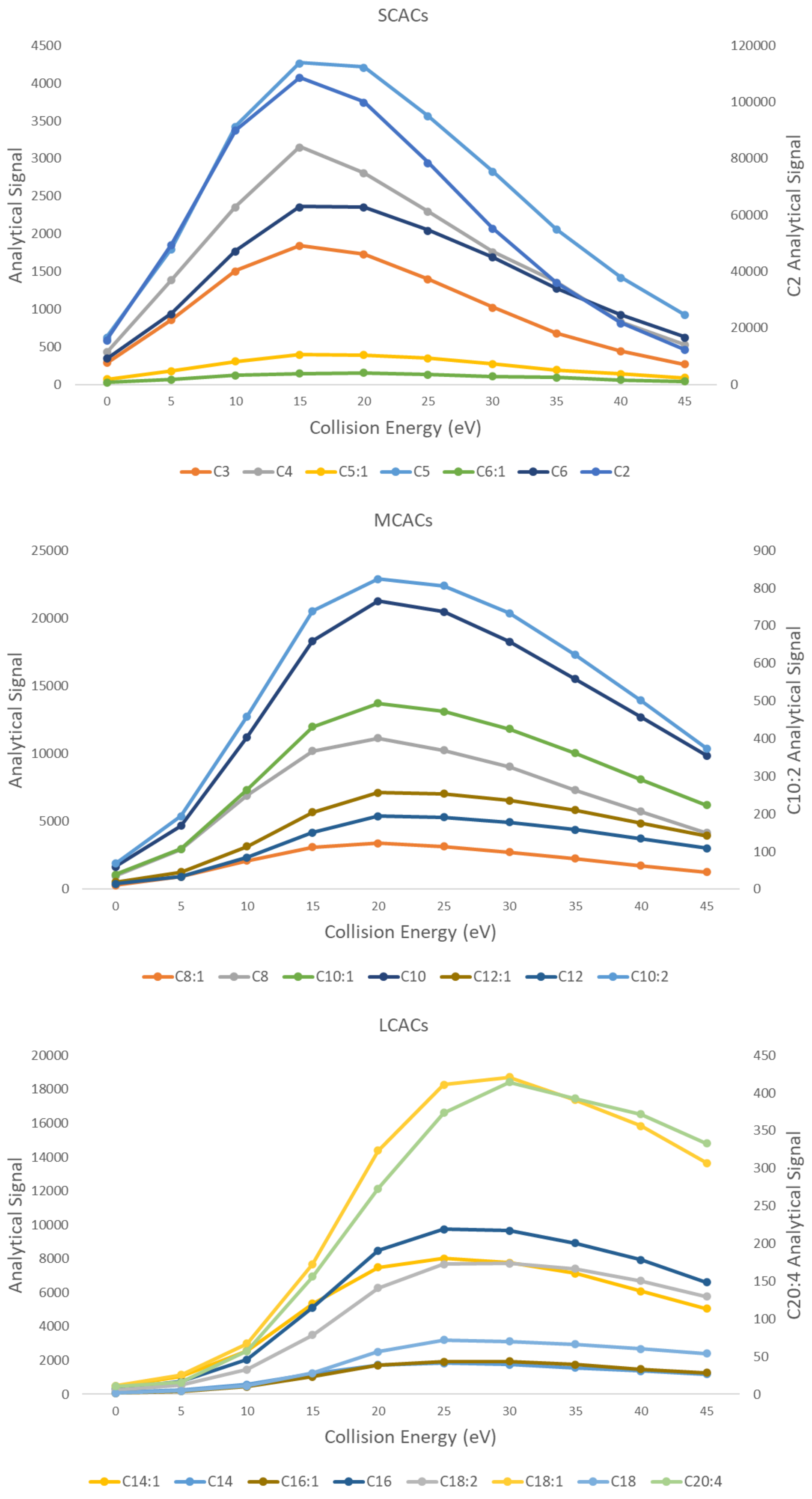

Fig. S3. Influence of the collision energy on the quantitative response of SCACs, MCACs and LCACs.

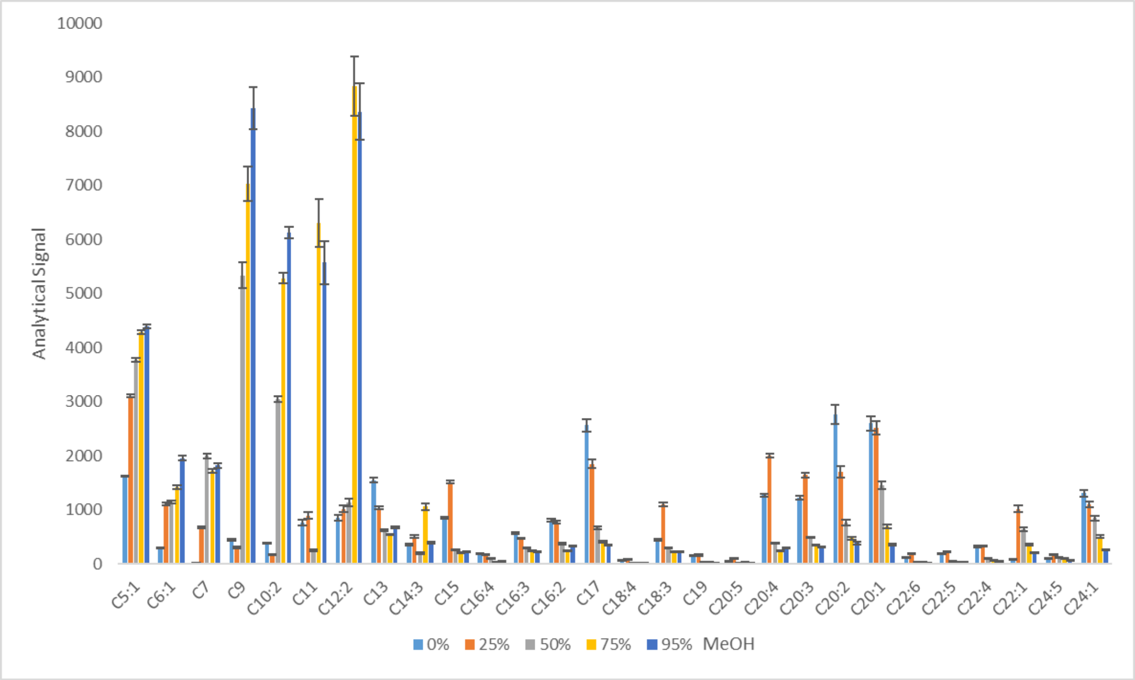

Fig. S4. Influence of the organic mobile phase composition on the ionization of the low-concentrated acylcarnitines, represented by MeOH concentration in phase B.

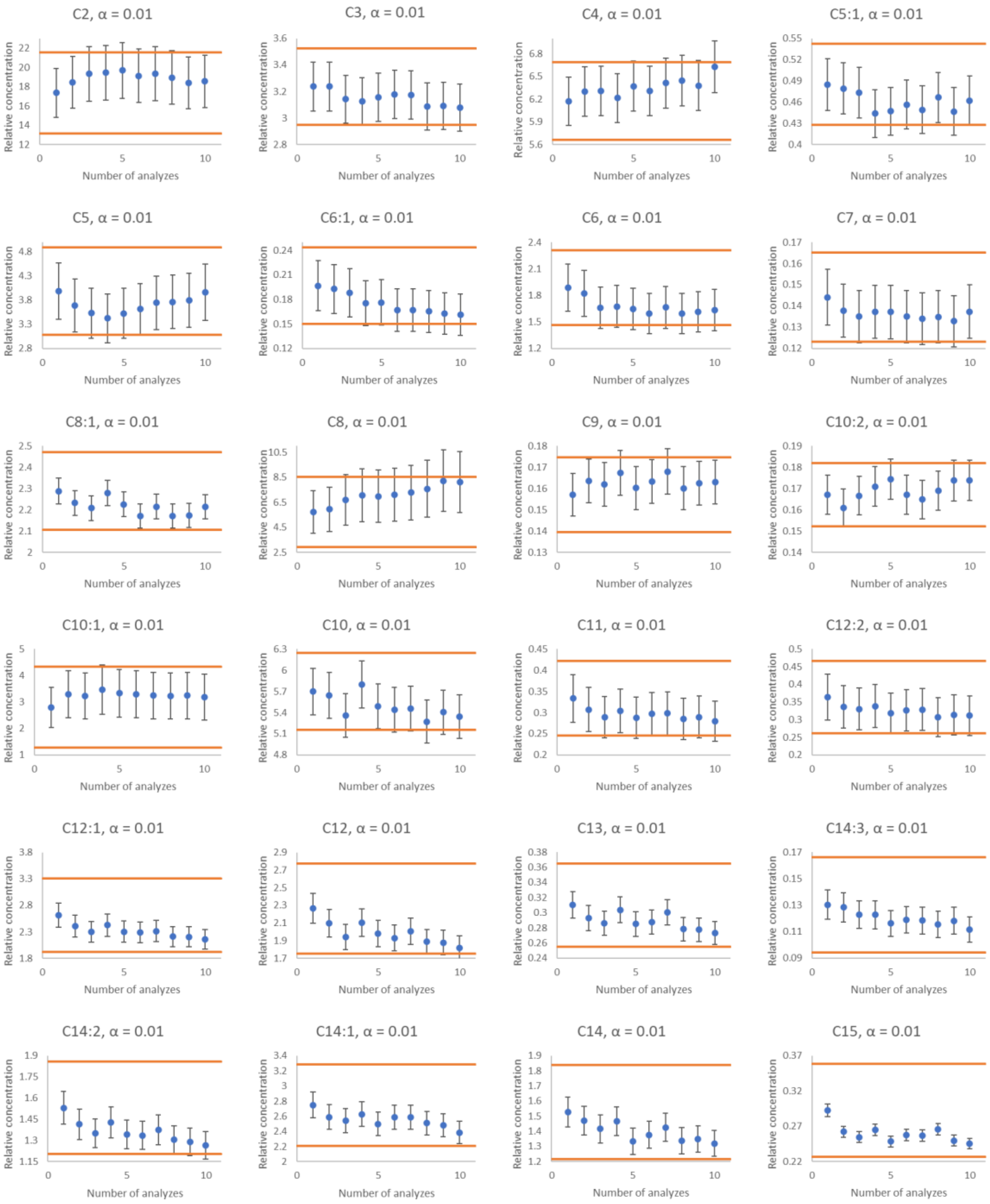
C2, α = 0.01
C3, α = 0.01
C4, α = 0.01
C5:1, α = 0.01
C5, α = 0.01
C6:1, α = 0.01
C6, α = 0.01
C7, α = 0.01
C8:1, α = 0.01
C8, α = 0.01
C9, α = 0.01
C10:2, α = 0.01
C10:1, α = 0.01
C10, α = 0.01
C11, α = 0.01
C12:2, α = 0.01
C12:1, α = 0.01
C12, α = 0.01
C13, α = 0.01
C14:3, α = 0.01
C14:2, α = 0.01
C14:1, α = 0.01
C14, α = 0.01
C15, α = 0.01
Relative concentration
Number of analyzes

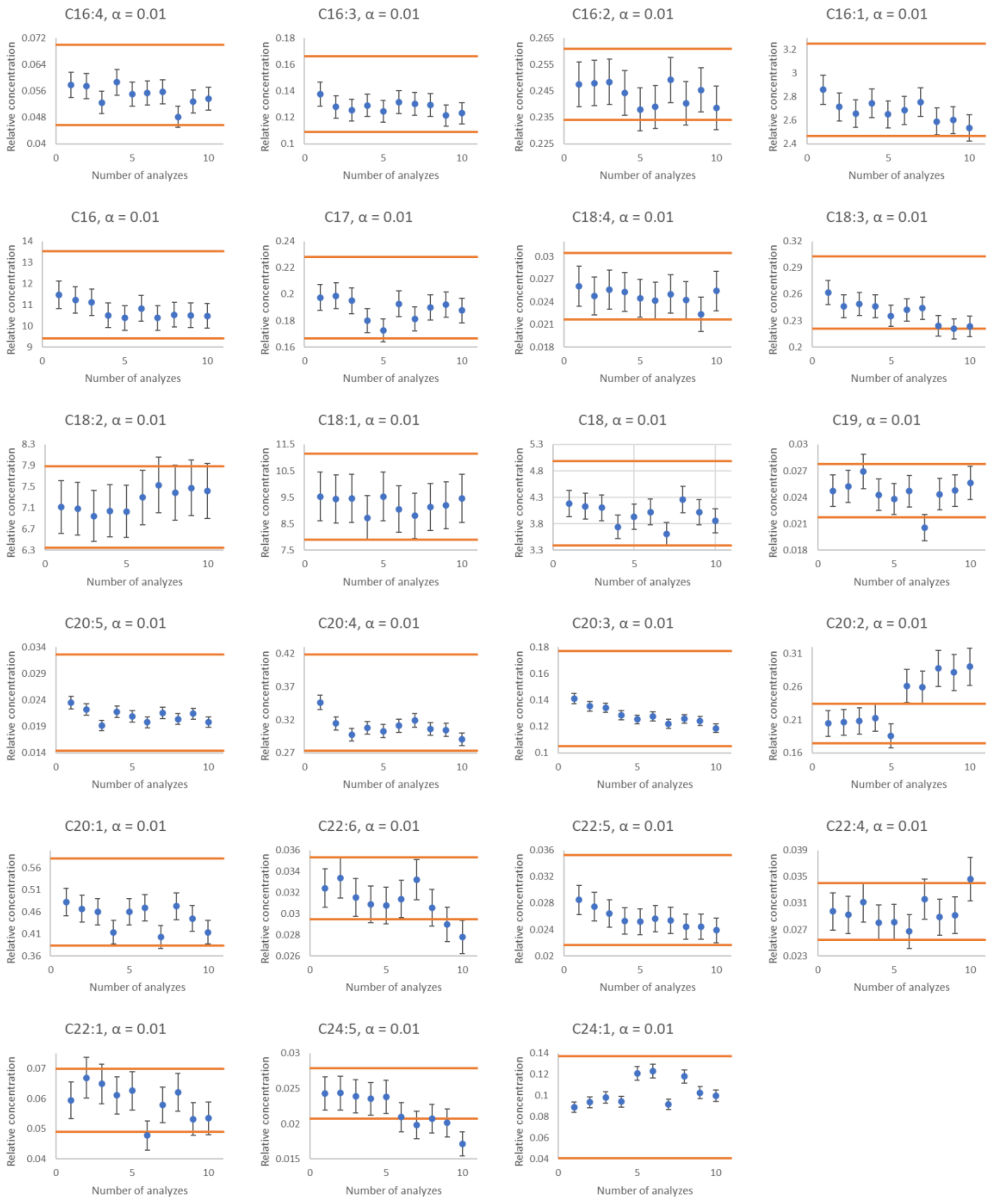

Fig. S5. Reusability test of the sorbent cartridge evaluated by 10 serum aliquots considering confidence intervals with significance level (α) 0.01.

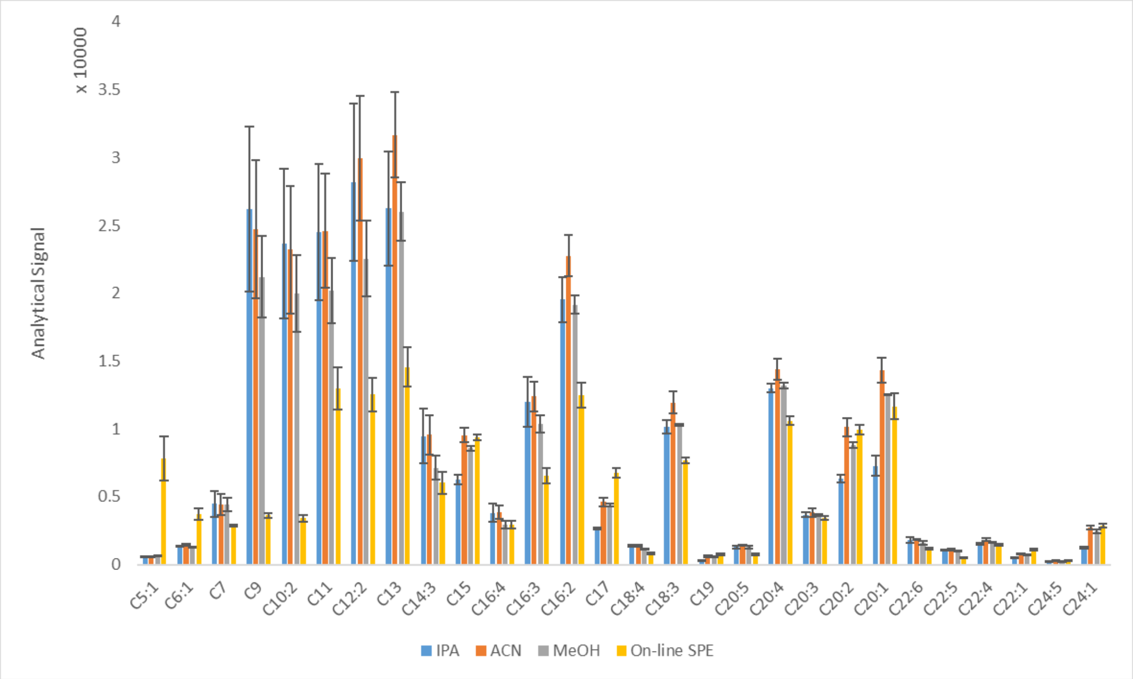

Fig. S6. Comparison of the quantitative signal for low-concentrated acylcarnitines provided by the proposed method and protein precipitation using different organic solvents (IPA, MeOH and ACN).

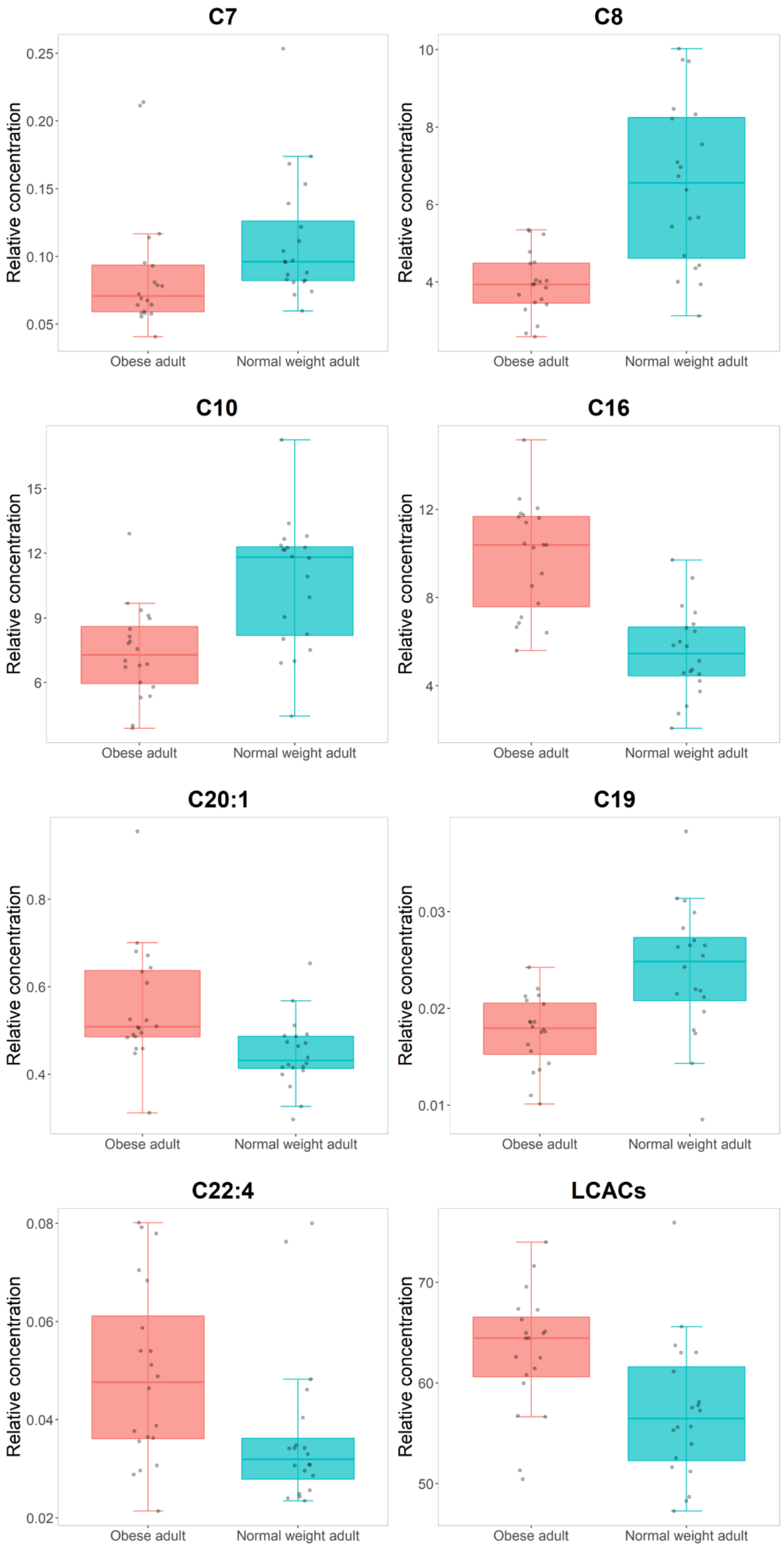

Fig. S7. Box-and-whisker plots presenting significant differences between normal weight and obese adults found in the relative concentration of 6 acylcarnitines and in the content of LCACs.

Bibliography

Psychogios, N., Hau, D. D., Peng, J., Guo, A. C., Mandal, R., Bouatra, S., Sinelnikov, I., Krishnamurthy, R., Eisner, R., Gautam, B., Young, N., Xia, J., Knox, C., Dong, E., Huang, P., Hollander, Z., Pedersen, T. L., Smith, S. R., Bamforth, F., … Wishart, D. S. (2011). The human serum metabolome. PLoS ONE, 6(2). https://doi.org/10.1371/journal.pone.0016957

Trabado, S., Al-Salameh, A., Croixmarie, V., Masson, P., Corruble, E., Fève, B., Colle, R., Ripoll, L., Walther, B., Boursier-Neyret, C., Werner, E., Becquemont, L., & Chanson, P. (2017). The human plasma-metabolome: Reference values in 800 French healthy volunteers; Impact of cholesterol, gender and age. PLoS ONE, 12(3), 1–17. https://doi.org/10.1371/journal.pone.0173615

Wang, M., Yang, R., Mu, H., Zeng, J., Zhang, T., Zhou, W., Wang, S., Tang, Y., Li, H., Zhang, C., Chen, W., & Dong, J. (2019). A simple and precise method for measurement of serum free carnitine and acylcarnitines by isotope dilution HILIC-ESI-MS/MS. International Journal of Mass Spectrometry, 446, 116208. https://doi.org/10.1016/j.ijms.2019.116208
